# Effects of drought and mycorrhiza on wheat and aphid infestation

**DOI:** 10.1002/ece3.6703

**Published:** 2020-08-28

**Authors:** Caroline Pons, Ann‐Cathrin Voß, Rabea Schweiger, Caroline Müller

**Affiliations:** ^1^ Department of Chemical Ecology Bielefeld University Bielefeld Germany

**Keywords:** aphids, arbuscular mycorrhiza, cereal, drought, phloem sap composition

## Abstract

The impacts of climate change on worldwide crop production become increasingly severe. Thus, sustainable enhancements of agricultural production are needed. The present study investigated the effects of drought and arbuscular mycorrhizal fungi on wheat plants (*Triticum aestivum*) and their interaction with aphids. Considering predicted climate change scenarios, wheat plants were exposed to well‐watered conditions, continuous drought (CD), or pulsed (PD) drought and plants were grown without (NM) or with mycorrhizal (AM) fungi. Ear biomass and harvest index were evaluated when grains were produced. Moreover, drought‐ and mycorrhiza‐induced changes in the amino acid composition of leaf phloem exudates were studied and the population growth and survival of *Sitobion avenae* aphids on those plants measured. Wheat plants responded differently toward the irrigation treatments. Under drought stress, ear biomass was reduced, while AM resulted in an enhanced harvest index. In phloem exudates especially, relative concentrations of the osmoprotectant proline were modulated by drought. Aphid population size was influenced by the interaction of drought and mycorrhiza treatment. This study emphasizes the pronounced influence of irrigation frequency on plant performance and indicates positive contributions of AM that may be relevant for agriculture.

## Graphical abstract

Wheat plants were exposed to different irrigation regimes, and the effects of arbuscular mycorrhiza (AM) on plant and aphid performance were tested. For plants experiencing continuous or pulsed drought, AM had a growth‐promoting effect. Aphids had a poorer survival on those plants, indicating positive effects of AM in agriculture.

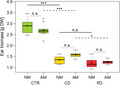

## 1 INTRODUCTION

Worldwide, crop cultivation is increasingly challenged as a result of climate change (Lobell & Gourdji, 2012). Enhanced CO_2_ levels and radiation lead to heatwaves and altered precipitations, exacerbating drought periods (Trenberth et al., 2014). Drought is defined as a climate event with below‐normal precipitation in relation to the local normal conditions (Dai, 2011), but can be differentiated into distinct types. Many studies investigate the constant shortage of precipitations, that is, continuous drought (CD) (Ding, Hayes, & Widhalm, 2011; Farooq, Wahid, Kobayashi, Fujita, & Basra, 2009). However, longer drought periods can be intermitted by heavy rainfall, which we here refer to as pulsed drought (PD). Such shifts between drought and heavy rainfalls are expected to increase in the future (IPCC, 2014; Trenberth et al., 2003). To secure the food supplies for a rising world population under current climate change scenarios, research on the consequences of different drought types for crop production and on potentials for plant growth promotion is essential.

For crop plants such as wheat (*Triticum aestivum* L., Poaceae), deficient water availability primarily leads to reduced aboveground biomass production (Gregorová et al., 2015; Nezhadahmadi, Prodhan, & Faruq, 2013), often resulting in lower yield (Al‐Karaki, McMichael, & Zak, 2004; Nakhforoosh, Grausgruber, Kaul, & Bodner, 2015). To cope with drought, water and nutrient uptake can be adjusted by prioritized investment in root rather than shoot biomass (Farooq et al., 2009). To maintain the turgor, osmolytes such as sugars and amino acids, in particular proline, are accumulated in plant cells (Singh, Kumar, Singh, Singh, & Prasad, 2015; Tatar & Gevrek, 2008). In flag leaves of wheat, various metabolites respond in distinct directions to different drought scenarios (Stallmann, Schweiger, Pons, & Müller, 2020). These changes emphasize that drought also has an impact on plant chemistry.

Desiccation does not only impair nutrient availability and transport within the plant but also alters other soil properties (Chaves, Maroco, & Pereira, 2003; Kuchenbuch, Claassen, & Jungk, 1986), all being important factors for plants. Beneficial soil organisms such as arbuscular mycorrhizal fungi (AMF) can improve plant water and nutrient supply and stabilize soil water‐stable aggregates (Parniske, 2008; Rillig, Wright, & Eviner, 2002). The relationship between AMF and plant roots, called arbuscular mycorrhiza (AM), is based on nutrient exchange. The fungus with its very fine hyphae acquires water and nitrogen‐ and phosphorus‐containing nutrients beyond the zone that can be accessed by the plant roots and delivers these compounds to the host plant (Cui & Caldwell, 1996; Khalvati, Hu, Mozafar, & Schmidhalter, 2005). In return, the plant provides the fungus with photoassimilates (Smith & Read, 2008). AM is found in over 80% of the terrestrial plant species (Smith & Read, 2008), and AMF are ubiquitous in various ecosystems (Parniske, 2008). The application of certain AMF species or communities as beneficial organisms for different agricultural crops is discussed (Rillig et al., 2019). Under drought, AM often enhances plant aboveground biomass compared to non‐mycorrhized (NM) plants (Jayne & Quigley, 2014). Furthermore, drought‐stressed plants with AM were shown to accumulate less proline in their tissues compared to NM plants (Augé, 2001; Wu, Zou, Rahman, Ni, & Wu, 2017), indicating an enhanced drought resistance of AM plants. Although grasses have a lower mycorrhizal response than dicots (Jayne & Quigley, 2014), AM was shown to promote the yield of wheat under well‐watered conditions (Pellegrino, Öpik, Bonari, & Ercoli, 2015; Zhang, Lehmann, Zheng, You, & Rillig, 2019) as well as under water deficiency (Al‐Karaki et al., 2004). However, to our knowledge it has not been investigated whether AM can buffer negative growth responses of wheat to different drought regimes.

Along with abiotic factors such as drought, crop plants often are exposed to biotic challenges such as herbivorous pests. Aphids feed on the phloem sap of their host plants and can transmit diseases (Trębicki et al., 2015). Due to their feeding style, aphids particularly depend on phloem sap traits, for example, with regard to water, nutrients, and osmotic pressure (Auclair, 1963). Drought‐stressed plants can be advantageous for aphids, because phloem sap feeders benefit from nitrogenous osmoprotectants such as free amino acids, as predicted by the “plant stress hypothesis” (White, 1969). More specifically, the “pulsed stress hypothesis” posits that the effect of watering with high amounts of water under PD can intensify the benefit for aphids, as turgor is recovered for a short period (Huberty & Denno, 2004). In contrast, the “plant vigour hypothesis” postulates that herbivores perform better on well‐watered, more nutritious plants or plant parts (Price, 1991). Given that aboveground plant chemistry is also altered by AM (Schweiger & Müller, 2015), this plant–fungus interaction likewise has an impact on herbivores (Koricheva, Gange, & Jones, 2009). Previous studies found positive, negative, or no influences of AM on aphid development, reproduction, and feeding behavior (Tomczak & Müller, 2017). Furthermore, the influence of AM on aphids changes over time along with the developmental stage of the AM and the host plants (Tomczak & Müller, 2017). Due to the complexity of the multidimensional interactions between drought, AMF, and aphids, it remains unclear how metabolic plant responses to different drought regimes may be modified by AM and how the plant status influences aphid performance.

In the present study, we investigated the influences of AMF and irrigation treatment, that is, well‐watered (CTR), CD, or PD, on various traits of wheat plants, including ear biomass, harvest index (HI = dry ear biomass/dry total aboveground biomass), and the amino acid composition of leaf phloem exudates. Moreover, we studied the consequences of these plant treatments on population development and survival of the English grain aphid, *Sitobion avenae* (Hemiptera: Aphididae), which is a common pest of wheat (Larsson, 2005; Vickerman & Wratten, 1979). We predicted a higher ear biomass and a higher HI in AM compared to NM plants particularly under drought stress conditions. Because both drought and AM are known to influence plant chemistry (Chaves et al., 2003; Farooq et al., 2009; Schweiger & Müller, 2015), we expected furthermore that the relative amino acid composition of leaf phloem exudates differs between plants of distinct treatments. In particular, the relative concentration of proline was predicted to be higher in phloem exudates of drought‐stressed plants, but within the drought‐stressed plants to be comparatively lower in AM plants due to improved water supply by AMF. For the aphids, we expected higher population sizes and longer survival of aphids on AM plants due to an improved nutrient uptake by these plants. Furthermore, we expected aphids to reproduce less and die faster on drought‐stressed NM plants as a consequence of the low water status compared to AM plants.

## 2 MATERIALS AND METHODS

### 2.1 Plant cultivation and mycorrhiza treatments

The experiment was carried out in a climate chamber at 22.7 ± 1.6°C (mean ± *SD*) with a relative humidity (r.h.) of 61 ± 5% and 16‐hr light:8‐hr dark. Untreated grains of the spring wheat cultivar Tybalt were provided by Borries‐Eckendorf. Plants were grown in 2‐L pots (11.3 × 11.3 × 23 cm) with a 2:1 mixture of steamed (4 hr, at 120°C) sand and soil (Fruhstorfer Pikiererde, Hawita Group). This mixture was used to be able to harvest and clean the roots at the end of the experiment to determine the total root length colonization (TRLC). The pots had holes at their base to enable draining of surplus water and were placed on holders to prohibit water loss. To pots of the arbuscular mycorrhizal treatment (AM; *n* = 30), 150 ml of fungal inoculum (sand spore mixture, pH 7; INOQ GmbH) was added, containing the generalist fungus *Rhizoglomus irregulare* (Błaszk., Wubet, Renker & Buscot) Sieverd., G. A. Silva & Oehl (Glomerales, Glomeromycota). This AMF species was used because it is commercially available for potential use in agriculture, colonizes wheat roots, and is known to affect growth and physiological responses of spring wheat (Campos et al., 2019; Zhang et al., 2018). The inoculum was mixed with the upper third of the substrate in each pot. To provide comparable microbial conditions in the non‐mycorrhized control treatment (NM; *n* = 30), 150 ml of sterilized (3 hr, at 120°C), inoculum was mixed with the upper third of the substrate of each pot, and 45 ml of a microbial solution was added, which was obtained from the inoculum before sterilization by filtrating a washing of the inoculum through a 20‐µm sieve. AM pots received 45 ml water instead. To simulate field conditions with regard to plant density and nutrient competition, each pot contained two plants in opposite corners, at a distance of about 10 cm. Sufficient germination was guaranteed by putting three seeds in the pots. Surplus seedlings were removed six days post sowing (dps). Plants were fertilized 18 (1 g/plant) and 35 dps (0.6 g/plant) with a solid long‐term, phosphate‐free mineral fertilizer (Floranid N‐P‐K 14‐0‐19, containing 3% Mg, 11% S, and traces of B, Cu, Fe, Mn, and Zn; Manna).

### 2.2 Establishment of irrigation treatments

**Figure 1:**
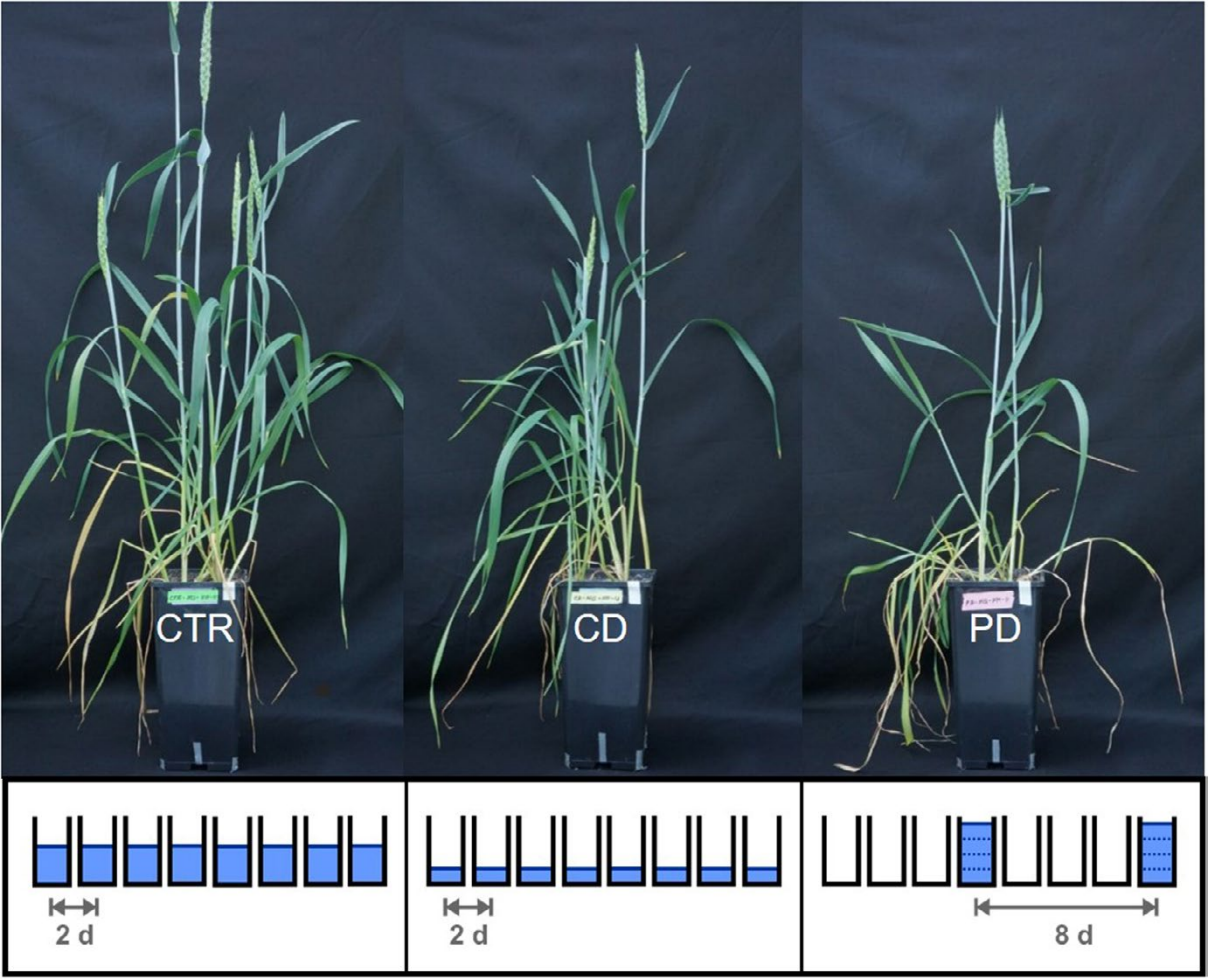
Photographs of wheat plants grown under different irrigation conditions [control (CTR), continuous drought (CD), and pulsed drought (PD)] at 51 days post sowing; the irrigation regimes are illustrated below the photographs

Initially, all pots were kept well‐watered near field capacity [~18% soil water content (SWC) determined in preliminary experiment] until 24 dps to gain robust plants. The water requirement was determined gravimetrically. Therefore, every other day 15 representative pots were chosen and weighed, and weights were averaged. Pots then received the calculated amount of water to regain the requested soil moisture. Within each mycorrhiza treatment (NM/AM), pots were randomly attributed at 24 dps to one of three irrigation treatments, a control (CTR), continuous drought stress (CD), or pulsed drought stress (PD) (*n* = 10 per mycorrhiza and irrigation treatment). CTR pots were continued to be weighed as described above and watered to 18% SWC. CD and PD pots were left unwatered until a SWC of ~8% was reached. Then, CD pots received 40% of the water amount that was added to the CTR pots. A reduction of precipitation to 40% may occur in certain hot and dry summers, especially in regions under ongoing climate change. Furthermore, this amount of water had been shown to result in a significantly reduced aboveground biomass of wheat in a previous study under similar growth conditions, without killing the plants (Stallmann, Schweiger, & Müller, 2018). CTR and CD plants were watered every other day. Pots of the PD treatment were irrigated only every eight days with the cumulated amount the CD pots had received in that time period (Figure 1). All pots were randomly distributed, and their position was changed weekly. The number of replicates was reduced by three pots (2 CD AM and 1 PD AM) due to a local fungal infection and by a further pot (CD NM) due to a failure during phloem exudate sampling. If not stated otherwise, in the following “treatment” is referred to as a combination of mycorrhizal treatment and irrigation treatment, for example, AM CTR.

### 2.3 Phloem exudate collection and plant harvest

To examine the influence of the different treatments on the phloem sap composition of developing wheat plants, phloem exudates were collected when ears were fully emerged and plants started flowering (52 dps, T1; Figure 1). At this time point, the aphid bioassays were started as well (see below). Phloem exudates were collected from half of the replicates (group A; *n* = 4–5 per treatment) from one plant per pot, following a method modified after Kos et al. (2011) and Schweiger, Heise, Persicke, and Müller (2014). To guarantee a sufficient turgor of the phloem sap, the phloem exudate collections took place 24 hr after watering all plants. The three youngest leaf blades of the main shoot were cut at their base and placed into a 50‐ml Falcon tube with 1 ml of an 8 mM ethylenediaminetetraacetic acid solution (EDTA; 99%, AppliChem GmbH; pH = 7, adjusted with NaOH) in the dark for 2 hr (20°C, 60% r.h.). After this first incubation, leaves were washed in Millipore water (MicroPure Water Purification System, Thermo Fisher Scientific), transferred to a new 50‐ml Falcon tube with 1 ml Millipore water, and incubated for another 2 hr in the dark. These second collections were used for subsequent chemical analyses. Blanks were prepared by keeping Millipore water without plant material in Falcon tubes for the same duration. For amino acid analysis, 300 µl of the exudates and blanks was frozen in liquid nitrogen and stored at −80°C. Leaf blades used for phloem exudate sampling were dried for 48 hr at 40°C and weighed.

When flowering was completed and grains were watery ripe (68 dps, T2), the total (remaining) aboveground plant biomass of both plants per pot was harvested for groups A and B. Biomass was separated into vegetative and generative (ears) parts and dried for 96 hr at 40°C to determine the dry biomass. The HI was calculated by dividing the dry ear biomass by the dry total aboveground biomass.

### 2.4 Amino acid analysis of phloem exudates

To analyze the amino acid composition, phloem exudates and blanks were lyophilized and redissolved in 80% methanol with norvaline and sarcosine (Agilent Technologies) as internal standards for primary and secondary amino acids, respectively. Samples were analyzed *via* high‐performance liquid chromatography coupled with fluorescence detection (HPLC‐FLD; 1260/1290 Infinity HPLC and FLD, Agilent Technologies) following the protocol of Jakobs and Müller (2018). Identification of amino acids was performed *via* comparison of retention times with those of reference standards measured within the same worklist. Amino acids were quantified by integrating the corresponding peaks, using OpenLab ChemStation C.01.07 (Agilent Technologies). Data were normalized by dividing the peak areas by the areas of the corresponding internal standards. Peak areas were related to the dry weight of the leaf blades from which the phloem exudates had been collected. Amino acids were only included into further analysis when they were found in at least three of the five replicates (or two of four replicates) of one treatment and not in the blanks. To compare the relative composition of amino acids in dependence of the plant treatment, for each amino acid, the mean percentage (i.e., its mean peak area divided by the mean total peak area of all amino acids) was determined for each treatment group. The amino acid data were only compared visually, because the sample sizes (*n* = 4–5) were too small for proper statistical analyses.

### 2.5 Root sampling and quantification of AMF colonization

To determine the TRLC with AMF, two subsamples of roots were taken per pot at T2 by punching a cork borer (2.8 cm i.d.) twice vertically into the substrate for about 22 cm. Subsequently, roots were washed and representative subsamples were bleached (10% KOH; 20 min, 95°C), dyed with an acetic solution of ink (royal blue, Ink 4001, Pelikan Group GmbH) (1:1:8 ink:acetic acid:water; 15 min, 90°C), and conserved (4:2:1 90% lactic acid:89% glycerin:water, 4°C in the dark). The TRLC was determined using the grid‐line intersect method (Giovanetti & Mosse, 1980) by separately counting different AMF structures (i. e., hyphae, vesicles, and arbuscules) in 200 intersects per sample.

### 2.6 Bioassays with aphids

Aphids of *S. avenae* were obtained from Koppert Biological Systems and kept in tents (58 × 58 × 58 cm) on wheat plants (cultivar: Tybalt) for several generations at 16‐hr light:8‐hr dark. To examine the influences of the plant treatments on aphid performance, we investigated the growth of aphid populations as well as the survival of single nymphs on leaves of the experimental plants over 16 days (from T1 to T2). To record the population growth, clip cages (2 cm i.d.) with three eight‐day‐old apterous aphids were attached to the second youngest leaf of the main shoot of one intact plant per pot (groups A and B, *n* = 8–10/treatment). Under the used conditions, aphids of *S. avenae* turn into adults at about 9 days and live for about 20–60 days. For group A, the plant in each pot was used that had not been used for phloem exudate collections (see above). Numbers of living adult aphids and their offspring were counted after 16 days on the plants (T2). To record the survival of single aphids, a one‐day‐old nymph was fixed in a clip cage on the flag leaf of the same shoot (*n* = 8–10/treatment). Survival of these nymphs was checked every day and offspring counted and removed.

### 2.7 Statistical analyses

All statistical analyses were carried out using R (R Core Team, 2019). Linear (mixed‐effects) models [L(M)M] were performed for responses with a normal error distribution and a generalized linear mixed‐effects model (GLMM) for the response with a Poisson distribution using the R package *lme4* (Bates et al., 2013). Model variance homogeneity and normal distribution of residuals were checked by visual inspection (Zuur, Ieno, Walker, Saveliev, & Smith, 2009), and type III analyses of variance based on chi‐square likelihood ratio tests were calculated afterward (R package *car*; Fox, Friendly, & Weisberg, 2013). All post hoc and contrast calculations were performed using the *glht* function in the R package *multcomp* (Hothorn, Bretz, & Westfall, 2008) and included *P* value corrections. To test the response of TRLC (arcsine‐ and square‐root‐transformed), a LM with the factorial predictor irrigation (CTR, CD, and PD) was performed with samples of the AM treatment group, followed by a post hoc Tukey test. The LMM for the responses ear dry biomass and HI comprised the factorial predictors mycorrhiza (NM and AM), irrigation (CTR, CD, and PD), and their interaction as well as the random effect of the harvest group (A, B). The GLMM for the response of aphid population size (Poisson distributed) at T2 comprised the factorial predictors mycorrhiza (NM and AM), irrigation (CTR, CD, and PD), and their interaction as well as the random effect of the harvest group (A, B). Manual contrasts were calculated to test for significant differences between irrigation groups within the same mycorrhiza group (e.g., CTR AM vs. CD AM) as well as between NM and AM within each irrigation group (e.g., CTR NM vs. CTR AM). To determine the influences of mycorrhiza, irrigation, and their interaction on the survival of single aphids, Kaplan–Meier survival curves (R package *survival;* Therneau & Lumley, 2015) were plotted and further analyzed with a mixed‐effects Cox regression model including the harvest group (A, B) as random factor (R package *coxme;* Therneau, 2018).

## 3 RESULTS

### 3.1 Mycorrhization

**Figure 2:**
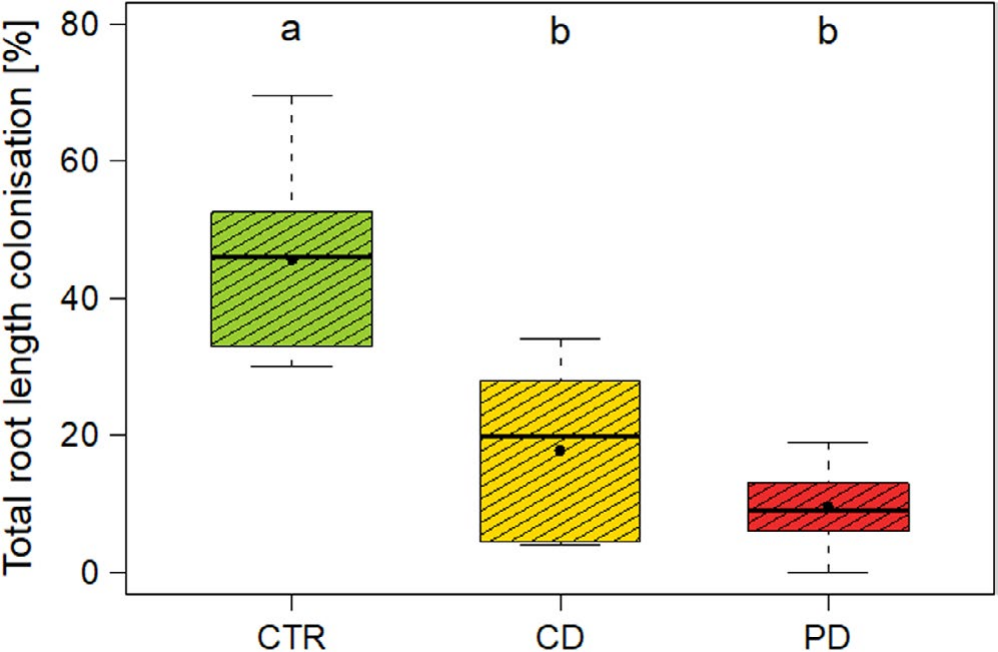
Total root length colonization of wheat (*Triticum aestivum*) roots 68 days post sowing (hyphae and vesicles; no arbuscules found). Plants were inoculated with the arbuscular mycorrhizal fungus *Rhizoglomus irregulare* and grown under different irrigation conditions [control (CTR), continuous drought (CD), and pulsed drought (PD)]. Data are given as box–whisker plots with interquartile ranges (IQR; boxes) including medians (horizontal lines), means (filled circles), and whiskers (extending to the most extreme data points with maximum 1.5 times the IQR). Different letters indicate significant differences between irrigation treatments according to a post hoc Tukey test; *n* = 8–10

The root colonization of AM plants was significantly influenced by irrigation (LM: *df* = 2, *X^2^* = 29.91, *p* < .001). The highest TRLC was determined in CTR plants (Figure 2) and was significantly different from CD and PD plants. In CD plants, the TRLC was on average 2.6 times lower and in PD plants 4.8 times lower compared to CTR roots; however, TRLC did not differ significantly between plants of the CD and PD treatment. Across all AM plants, the main intraradical mycorrhizal structures were hyphae (CTR plants: 63.1% ± 21.5%; CD plants: 66.1% ± 27.7%; PD plants 68.9% ± 35.9%; mean ± *SD*), followed by vesicles (CTR plants: 36.9% ± 21.5%; CD plants 33.9% ± 22.7%; PD plants: 31.3% ± 29.5%; in two of the PD plants, no vesicles were found), whereas arbuscules were not detected. None of the NM plants showed mycorrhizal structures.

### 3.2 Ear biomass and harvest index

**Figure 3:**
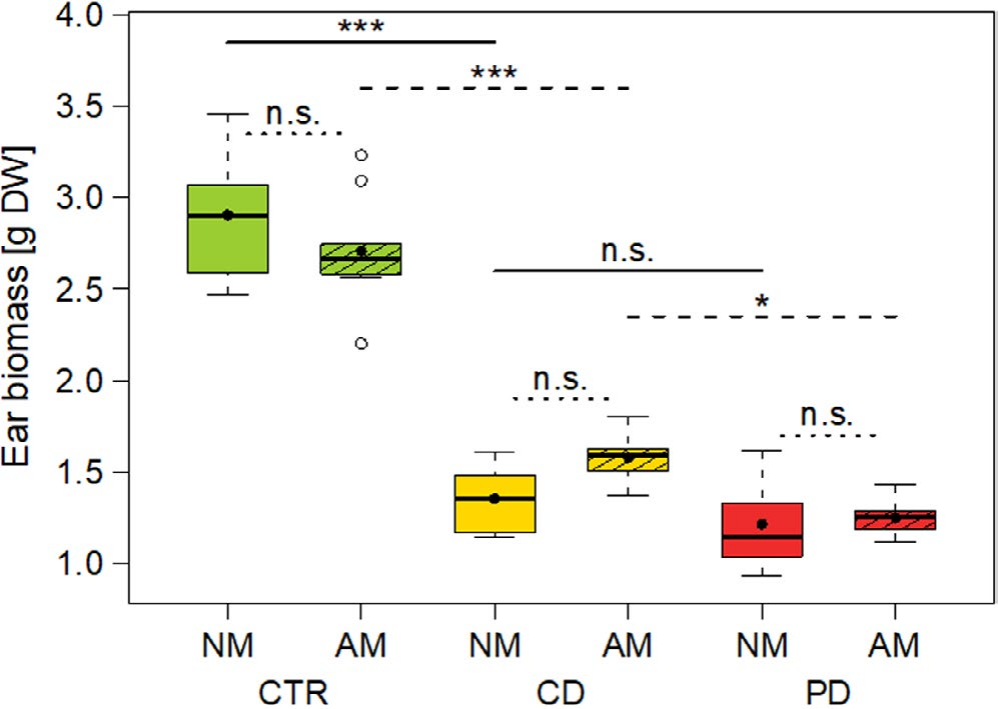
Dry ear biomass (68 days post sowing) of wheat (*Triticum aestivum*) plants either non‐mycorrhized (NM) or mycorrhized (AM) with *Rhizoglomus irregulare*. Plants were grown under different irrigation conditions [control (CTR), continuous drought (CD), and pulsed drought (PD)]. Data are given as box–whisker plots with interquartile ranges (IQR; boxes) including medians (horizontal lines), means (filled circles), and whiskers (extending to the most extreme data points with maximum 1.5 times the IQR); outliers are given as open circles. Results of manual contrasts for selected pairwise group comparisons are given between different irrigation treatments within NM (solid lines)/AM (dashed lines), and between NM and AM within each irrigation treatment (dotted lines); n.s., not significant (*p* > .05), *p < .05, ***p < .001; *n* = 8–10

**Figure 4:**
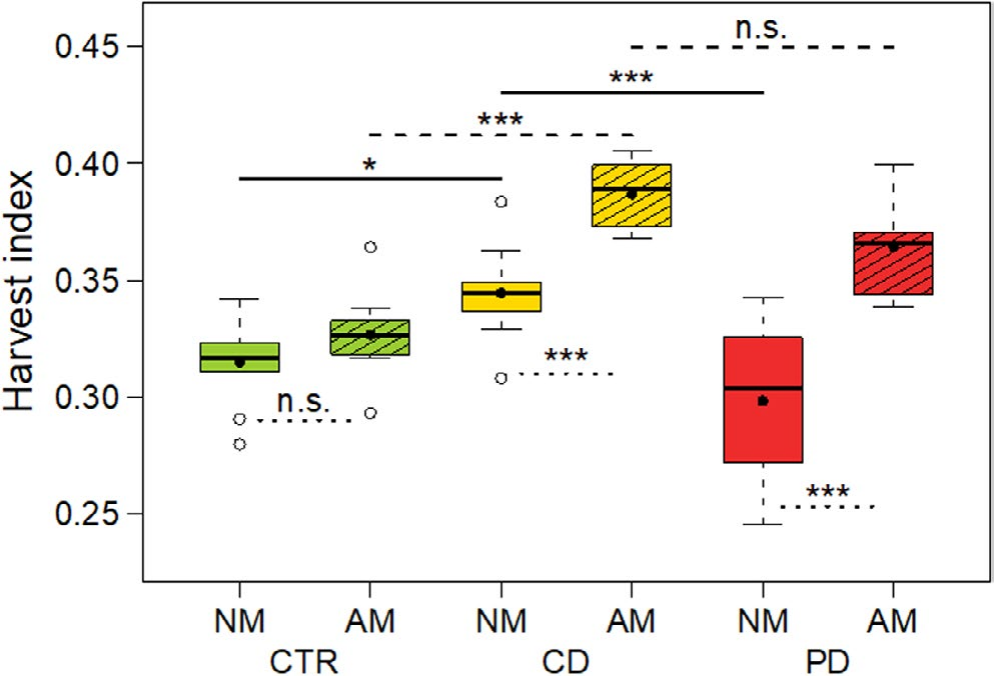
Harvest index (dry ear biomass/dry total aboveground biomass; 68 days post sowing) of wheat (*Triticum aestivum*) plants either non‐mycorrhized (NM) or mycorrhized (AM) with *Rhizoglomus irregulare*. Plants were grown under different irrigation conditions [control (CTR), continuous drought (CD), and pulsed drought (PD)]. Data are given as box–whisker plots with interquartile ranges (IQR; boxes) including medians (horizontal lines), means (filled circles), and whiskers (extending to the most extreme data points with maximum 1.5 times the IQR); outliers are given as open circles. Results of manual contrasts for selected pairwise group comparisons are given between different irrigation treatments within NM (solid lines)/AM (dashed lines), and between NM and AM within each irrigation treatment (dotted lines); n.s., not significant (*p* > .05), *p < .05, ***p < .001; *n* = 8–10

The dry ear biomass at T2 was significantly influenced by the interaction irrigation × mycorrhiza treatment (LMM: *df* = 2, *X^2^* = 8.26, *p* = .016). CTR plants had a nearly two times higher ear biomass than drought‐stressed plants, with PD plants showing the lowest ear biomass (Figure 3). There were no significant differences in ear biomass between NM and AM plants within any irrigation group. Within drought‐stressed plants, the ear biomass was significantly higher in CD AM plants compared to PD AM plants. For the HI, the interaction irrigation × mycorrhiza treatment was likewise highly significant (LMM: *df* = 2, *X^2^* = 15.72, *p* < .001). The HI was overall highest in CD plants and was significantly enhanced by AM in the plants of both drought treatments (Figure 4).

### 3.3 Amino acid composition of phloem exudates

**Figure 5:**
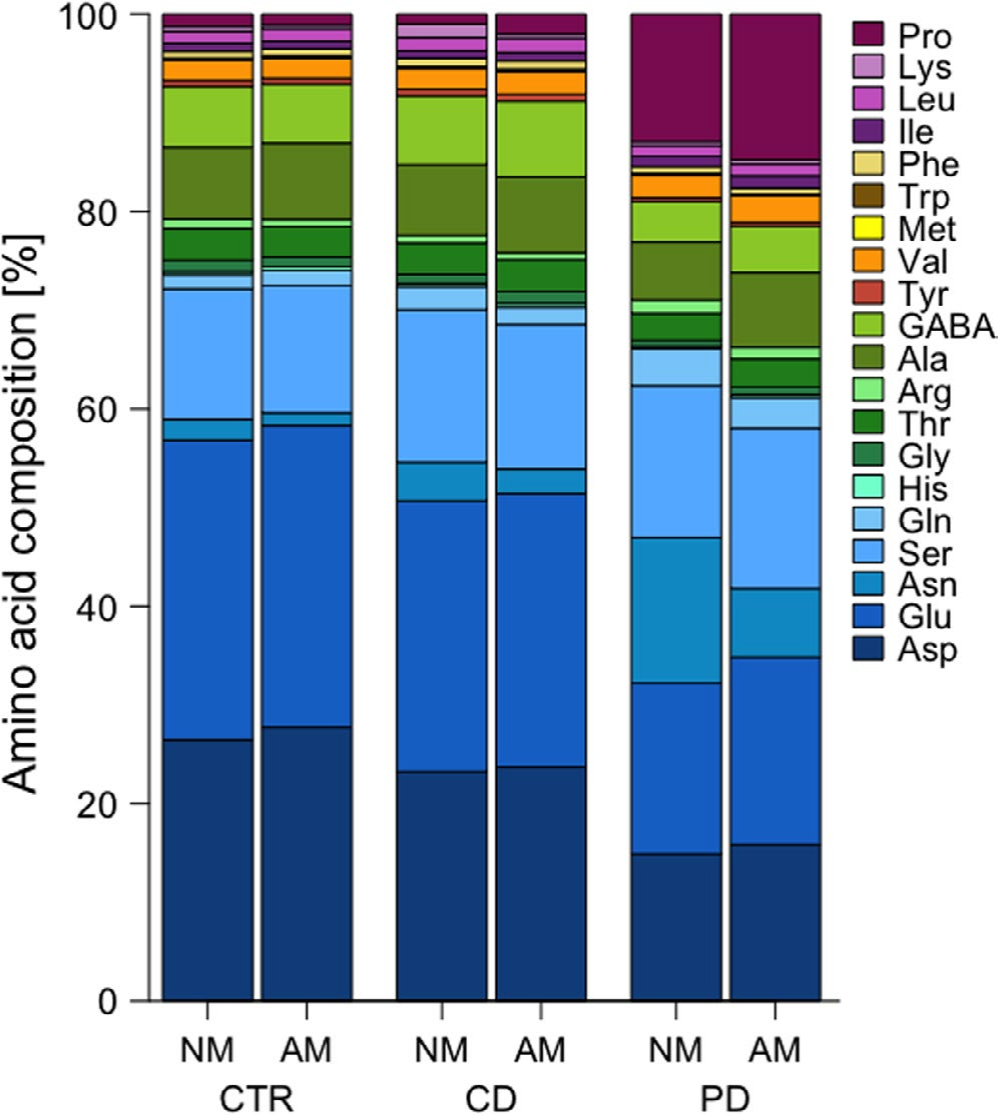
Relative concentrations of amino acids in phloem exudates of wheat (*Triticum aestivum*) plants either non‐mycorrhized (NM) or mycorrhized (AM) with *Rhizoglomus irregulare*. Plants were grown under different irrigation conditions [control (CTR), continuous drought (CD), and pulsed drought (PD)]. Exudates were collected *via* the EDTA method from the three youngest leaf blades of the main shoot at 52 days post sowing. Amino acid names are given in the common three‐letter code; GABA stands for γ‐aminobutyric acid. Means of *n* = 4–5 replicates per treatment

In total, 20 amino acids were retained in the data set. The amino acid composition of leaf phloem exudates differed between plants of different treatments (Figure 5). In particular, the relative concentration of proline was around nine times higher, while the proportion of glutamic acid was about 36% lower in PD compared to CTR and CD plants. No clear differences were found in these and other amino acids between NM and AM plants.

### 3.4 Aphid populations and survival

**Figure 6:**
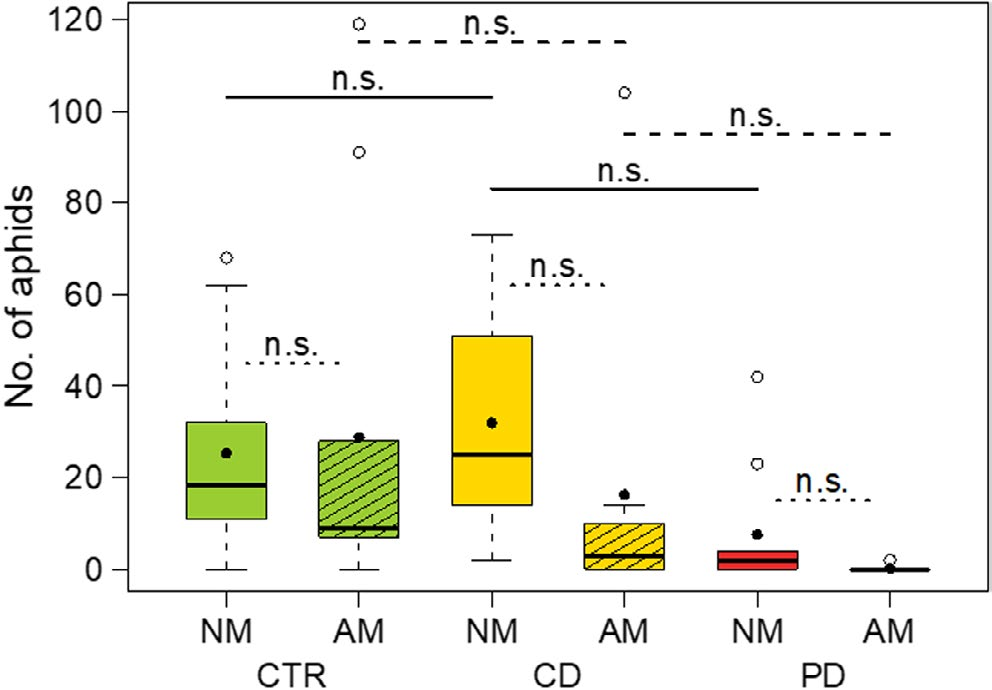
Population sizes of aphids (*Sitobion avenae*) after 16 days on wheat (*Triticum aestivum*) plants. Clip cages were attached at 52 days post sowing on the second youngest leaf of plants either non‐mycorrhized (NM) or mycorrhized (AM) with *Rhizoglomus irregulare*. Plants were grown under different irrigation conditions [control (CTR), continuous drought (CD), and pulsed drought (PD). Data are given as box–whisker plots with interquartile ranges (IQR; boxes) including medians (horizontal lines), means (filled circles), and whiskers (extending to the most extreme data points with maximum 1.5 times the IQR); outliers are given as open circles. Results of manual contrasts for selected pairwise group comparisons are given between different irrigation treatments within NM (solid lines)/AM (dashed lines), and between NM and AM within each irrigation treatment (dotted lines); n.s., not significant (*p* > .05); *n* = 8–10

**Figure 7:**
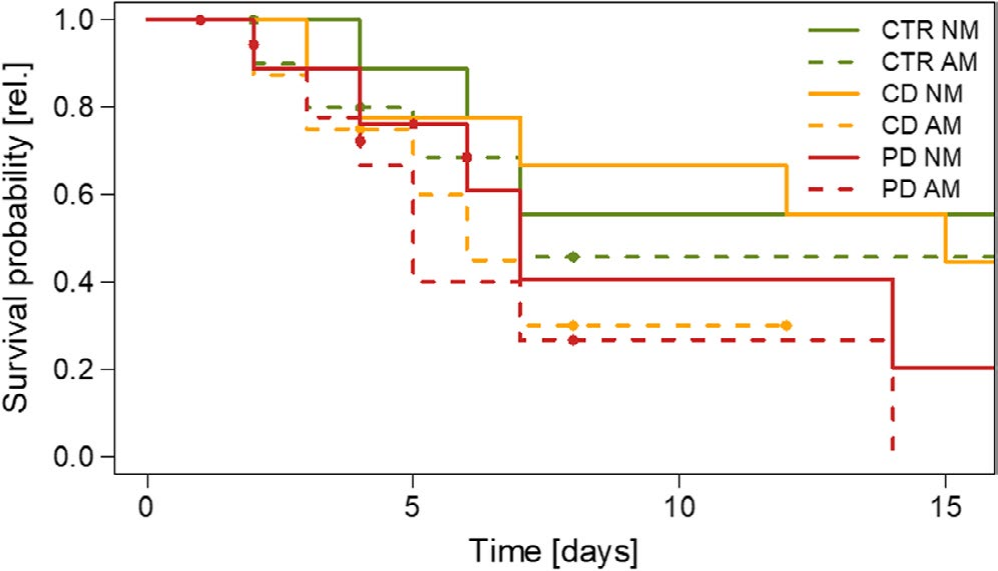
Kaplan–Meier survival curves of single aphids (*Sitobion avenae*) on wheat (*Triticum aestivum*) plants. Clip cages were attached at 52 days post sowing for 16 days on flag leaves of plants either non‐mycorrhized (NM) or mycorrhized (AM) with *Rhizoglomus irregulare*. Plants were grown under different irrigation conditions [control (CTR), continuous drought (CD), and pulsed drought (PD). Aphid survival was recorded every day. Filled circles indicate censoring before termination of the experiment; *n* = 8–10

The number of aphid individuals per population at T2 was significantly influenced by the interaction irrigation × mycorrhiza treatment (GLMM: *df* = 2, *X^2^* = 99.97, *p* < .001). On CD NM plants, aphid populations grew biggest followed by CTR NM and CTR AM plants (Figure 6). In contrast, populations shrank on PD NM and in particular on PD AM plants. Many aphid populations on CD AM (50%), PD NM (40%), and PD AM (89%) plants had already died before the assessment of population sizes after 16 days. Neither irrigation (mixed‐effects Cox regression model, *df* = 2, *X^2^* = 3.07, *p* = .215) nor mycorrhiza treatment (*df* = 1, *X^2^* = 3.27, *p* = .070) nor their interaction (*df* = 2, *X^2^* = 0.14, *p* = .934) had a significant influence on the survival of single aphids. However, the survival of single aphids on the flag leaves (Figure 7) reflected mostly the development pattern of the aphid populations (Figure 6). After 16 days, more than 40% of the single aphids were still alive on CTR plants and CD NM plants, while survival was lower on CD AM and PD plants. The number of offspring produced by the single aphids until day 16 also resembled the patterns described for population sizes at day 16 and survival over time, being lowest in PD and CD AM plants (data not shown).

## 4 DISCUSSION

In our experiment, both CD and PD stress resulted in a substantially reduced ear dry biomass. Water shortage often leads to reduced plant growth, because the nutrient uptake and transport are impaired by low soil moisture and cell elongation is impaired by low turgor (Chaves et al., 2003; Farooq et al., 2009). Furthermore, under drought, stomata usually close and hence transpiration and photosynthesis are reduced (Ashraf & Harris, 2013; Farooq et al., 2009; Xu, Zhou, & Shimizu, 2010). Here, we show that not only water amounts are responsible for plant growth, but irrigation frequency also plays an important role. Although receiving overall the same amount of water, the PD treatment caused a different and more severe stress than CD, as indicated by the lowest ear biomass in PD plants. While pulsed‐watered plants showed also lower grain yields than continuously watered wheat plants in a comparable study, the vegetative shoot dry biomass did not differ between plants of those groups but declined with decreasing irrigation volume (Stallmann et al., 2018). CD plants may be more used to the constant water shortage and able to adjust their water balance more effectively (Boyle, McAinsh, & Dodd, 2016). In contrast, watering of PD plants with larger amounts of water but at lower frequency forces these plants to newly tailor their water balance in every watering cycle leading to inefficient drought adjustment (Boyle et al., 2016).

Root colonization by AMF was lower in drought‐stressed plants compared to CTR plants, which is consistent with other studies on wheat (Al‐Karaki et al., 2004; Beltrano & Ronco, 2008). Under the PD condition, formation of mycorrhizal structures in the roots was particularly low, as also reported by Beltrano and Ronco (2008) for wheat under severe drought stress. The germination of AMF spores and development of AM structures are initiated based on plant signals in root exudates (Besserer et al., 2006) and may be impaired under low soil moisture. In the present experiment, probably the AMF germinated and AM was established during the well‐watered conditions in all plants (0–24 dps). During the subsequently reduced irrigation, the CD and PD plants may not have been able to provide the mycobiont with as many photoassimilates as the CTR plants, leading to a suppressed AMF colonization and in some PD plants a lack of formation of vesicles with storage function. Arbuscules were probably formed and functional during earlier stages of the plant–AMF interaction, explaining the AM effects on HI observed, but, due to their rapid turnover, may not have been visible at root harvest (T2). In contrast to our expectation, AM did not significantly increase the ear biomass in CD and PD plants. Nevertheless, under drought, the HI was, as hypothesized, positively influenced by AM, whereas CTR plants showed no difference in HI between AM and NM plants. This result shows that under drought stress, AM leads to a preferential relative investment of resources into reproductive parts (ears) rather than into vegetative biomass (shoots and leaves). As a consequence, for the wheat variety used in this experiment, growth‐promoting effects of AM may become more apparent under drought stress conditions.

A growth‐promoting effect of AM on plant aboveground biomass, or as in our case on the HI, may result from several mechanisms. AMF are able to improve the soil structure by enhancing the soil aggregate water stability, for example, by excretion of glomalin (Ji, Tan, & Chen, 2019; Rillig et al., 2002). Due to improved soil properties and the fine mycelium of the fungi, the AMF mobilize water and nutrients otherwise inaccessible for plant roots (Khalvati et al., 2005; Wilkinson, Ferrari, Hartley, & Hodge, 2019). Moreover, AM can increase the stomatal conductance in shoots and thereby enhance photosynthesis particularly under drought conditions (Augé, 2000; Augé, Toler, & Saxton, 2015). For example, AM plants of maize (*Zea mays* L., Poaceae) under water deficiency showed an increased stomatal conductance as well as plant biomass (Quiroga et al., 2019). It is well‐known that plant species, cultivars, and accessions respond differently to AMF. Many forbs show a high growth dependency on AM, while the growth response of grasses to AM varies from positive over neutral to negative effects (Hetrick, Wilson, & Cox, 1993; Höpfner, Friede, Unger, & Beyschlag, 2015; Plenchette, Fortin, & Furlan, 1983; Watts‐Williams et al., 2019). Our results indicate that the responsiveness may also differ between plant organs, leading to distinct investments of resources into different plant parts.

In addition to morphological alterations, plants also respond on a metabolic level to factors such as drought (Farooq et al., 2009; Gregorová et al., 2015; Shanker et al., 2014) or AM (Schweiger & Müller, 2015). As postulated, the relative composition of amino acids in the phloem exudates varied between plants of the different treatments. Particularly, proline had high relative concentrations in plants of the PD treatment. Proline is known to be involved in plant responses to drought and salinity (Singh et al., 2015). Under drought, proline functions as an osmolyte but also as osmoprotectant, for example, against reactive oxygen species in order to prevent protein damage (Szabados & Savouré, 2010). As osmoregulator, proline acts at the cellular level (Singh et al., 2015) but is usually measured as absolute concentration in leaves or other plant parts (Khosravifar, Farahvash, Aliasgharzad, Yarnia, & Khoei, 2020; Wu et al., 2017). Little is known about relative proline concentrations in the phloem sap. We argue that a change in the proportion of this amino acid in the phloem sap may likewise be a clear indicator of plant responses to drought. The negative relationship between proportions of proline versus glutamic acid in the phloem exudates of PD plants may be related to their tight biosynthetic relationship. In plants, proline is mainly synthesized from glutamic acid *via* some intermediates, and proline catabolism yields glutamic acid (Kavi Kishor et al., 2005; Szabados & Savouré, 2010). The shifts in proline and glutamic acid proportions in PD plants in our study may be due to an altered regulation of the corresponding enzymatic steps. Indeed, a higher proline synthesis and reduced catabolism have been found in other drought‐stressed plants (Kavi Kishor et al., 2005; Szabados & Savouré, 2010).

No pronounced differences were found in the relative amino acid composition of phloem exudates from NM versus AM plants. Similarly, in five plant species of different relatedness, the absolute foliar concentrations of most amino acids were not affected by AM (Schweiger, Baier, Persicke, & Müller, 2014). However, lower absolute concentrations of proline in leaves of AM compared to NM plants have been reported from trifoliate orange (*Poncirus trifoliata* L., Rutaceae) (Wu et al., 2017), while enhanced concentrations of glutamic acid were found in AM plants of sorghum (*Sorghum bicolor* L., Poaceae) (Abdel‐Fattah & Mohamedin, 2000) and strawberry (*Fragaria* × *ananassa* Duch., Rosaceae) (Matsubara, Ishigaki, & Koshikawa, 2009). The responses seem to be highly plant species‐ and treatment‐specific but may also differ on the leaf compared to the phloem sap level. In addition, AM effects on plant metabolites may change related to the establishment of AM along with plant development (Schweiger, Baier, & Müller, 2014), which may explain contrasting findings.

Population growth of *S. avenae* was affected by the irrigation treatment in interaction with AM, with lowest population sizes in CD AM and all PD plants. This finding emphasizes that both irrigation frequency and AM are not only important for plants but also for their herbivores. Regarding the plant vigor hypothesis (Price, 1991), the plant stress hypothesis (White, 1969), and the pulsed stress hypothesis (Huberty & Denno, 2004), our findings of aphid development on drought‐stressed compared to CTR plants stand between these hypotheses, because we did not find significant differences between aphid population development and survival in pairwise comparisons between treatments. For phloem‐feeding herbivores, the water status of their host plant is important in terms of plant tissue structure and cell turgor. Well‐watered plants should maintain a constant turgor allowing aphids to reach and ingest the phloem sap readily. In contrast, tissues of PD plants may be less stable, offering aphids only a chance to feed shortly after watering until turgor decreases again. Such results were found for *Brevicoryne brassicae* L. and *Myzus persicae* Sulzer, which reproduced better on plants of *Brassica oleracea* (Brassicaceae) under constant medium drought than on control plants or plants experiencing severe or pulsed drought (Tariq, Wright, Rossiter, & Staley, 2012). Aphids of *S. avenae* on *T. aestivum* plants under drought stress (10% SWC) had a reduced performance compared to those on non‐stressed plants (20% SWC), which may have been related to an upregulation of phytohormone‐regulated defense responses in the plants (Xie et al., 2020).

Against our expectation, *S. avenae* aphids did neither built up a larger population size nor survived longer on AM compared to NM plants. Although the effect was not significant, populations on AM CD plants were on average smaller than on the corresponding NM plants and less populations survived on AM PD compared to NM PD plants. Thus, the postulated benefit for aphids resulting from an ameliorated nutrient supply or water status due to AMF cannot be supported with our data. Overall, negative effects of AM on aphids have been less often found than positive ones (Koricheva et al., 2009; Tomczak & Müller, 2017).

The metabolic composition of the phloem sap is crucial for aphids. Plant phloem sap is a challenging diet, as sugar concentrations are usually very high leading to osmotic challenges, whereas the concentrations of (particularly essential) amino acids are low. In order to maintain turgor under drought, plants accumulate sugars (Farooq et al., 2009). Furthermore, in drought‐stressed AM plants, the concentration of sugars can be higher than in drought‐stressed NM plants (Wu et al., 2017). Consequently, instead of being more nutritious, drought‐stressed AM plants, especially under the more severe pulsed drought stress, may be of poor quality to aphids due to low water status and high sugar concentrations. Other important factors altering the host plant quality are specialized metabolites, which could be affected in concentrations by drought and AM (Akula & Ravishankar, 2011; Schweiger & Müller, 2015). On AM plants of the wheat species *T. aestivum* and *T. monococcum*, aphids fed longer, grew, and reproduced more than on NM plants, regardless of plant susceptibility to aphids, possibly because AM plants showed an increased width of vascular bundles (Simon, Wellham, Aradottir, & Gange, 2017). However, in the present study, the slightly negative influence of AM on aphid population sizes may have been a result of aggravated phloem uptake or even starvation caused by an increased phloem sugar content, which can result from drought (Farooq et al., 2009; Singh et al., 2015) and AM (Wu et al., 2017). It has to be further investigated whether changes in the proportions of proline and glutamic acid as observed in the current study can affect aphids. Next to optimum curves for individual amino acids regarding aphid performance, the ratios of different amino acids are probably crucial for efficient protein biosynthesis by aphids. Interestingly, an infestation of wheat by *S. avenae* can also cause changes in the amino acid composition as well as in the ratio of essential to non‐essential amino acids and the ratio of amino acids to sugars in phloem exudates (Liu et al., 2020). More research is needed to uncover the exact mechanisms that result in different aphid responses to stress‐exposed plants and to determine whether plant responses to aphid infestation are modified by various interfering abiotic stresses. The findings of previous studies and those from the presented one emphasize the complexity of plant–drought–mycorrhiza–aphid interactions.

## 5 CONCLUSIONS

The results of this study contribute another puzzle piece to understanding the responses of wheat toward insufficient irrigation and the role of AM in this system. We showed that AM can improve the HI of wheat plants under different drought scenarios and that aphid pests show a slightly reduced performance on AM plants. Thus, application of AMF may be highly advantageous in agriculture (Rillig et al., 2019), particularly under the predicted climate change scenarios.

## CONFLICT OF INTEREST

The authors declare no competing interests.

## AUTHOR CONTRIBUTION

**Caroline Pons:** Conceptualization (equal); Data curation (equal); Formal analysis (equal); Investigation (lead); Visualization (lead); Writing‐original draft (lead). **Ann‐Cathrin Voß:** Formal analysis (supporting); Investigation (supporting). **Rabea Schweiger:** Validation (equal); Writing‐review & editing (equal). **Müller Caroline:** Conceptualization (equal); Data curation (equal); Funding acquisition (lead); Project administration (lead); Supervision (lead); Validation (equal); Writing‐review & editing (lead).

## Data Availability

The data of this manuscript are available in Dryad, https://doi.org/10.5061/dryad.05qfttf13
